# Reducing the impact of geometric errors in flow computations using velocity measurements

**DOI:** 10.1002/cnm.3203

**Published:** 2019-04-16

**Authors:** David Nolte, Cristóbal Bertoglio

**Affiliations:** ^1^ Bernoulli Institute University of Groningen Groningen Netherlands; ^2^ Center of Mathematical Modeling University of Chile Santiago Chile

## Abstract

Numerical blood flow simulations are typically set up from anatomical medical images and calibrated using velocity measurements. However, the accuracy of the computational geometry itself is limited by the resolution of the anatomical image. We first show that applying standard no‐slip boundary conditions on inaccurately extracted boundaries can cause large errors in the results, in particular the pressure gradient. In this work, we therefore propose to augment the flow model calibration by slip/transpiration boundary conditions, whose parameters are then estimated using velocity measurements. Numerical experiments show that this methodology can considerably improve the accuracy of the estimated pressure gradients and 3D velocity fields when the vessel geometry is uncertain.

## 1 INTRODUCTION

The pressure drop (PD) across stenotic blood vessels is a standard clinical diagnostic quantity. It is used to assess the severity of the pathology and to stratify patients for therapy. Examples include the use of PD in aortic coarctation patients,^1^ cases of valve stenosis,^2^, ^3^ and congenital heart diseases.^4^ The current gold standard procedure in the clinical practice for measuring the PD is invasive X‐ray guided pressure catheterization. However, due to the invasive nature of the method, it is often preferred to estimate the PD from noninvasive velocity measurements. In the clinical practice, PD is most often computed from Doppler echocardiography^5^ by using a simplified Bernoulli equation, which however does not take the particular patient's anatomy into account.

Phase‐Contrast Magnetic Resonance Imaging (PC‐MRI) permits noninvasive, time‐ and space‐resolved measurements of the blood flow velocity in anatomically complex regions, either in selected planes (2D)^6^ or in the whole thorax (3D).^7^ PC‐MRI allows measuring the velocity field with spatial and temporal resolutions in the range of 1 to 3 mm and 20 to 40 ms, respectively, and noise levels of around 15 % of the maximal velocity.^8^ The ability of PC‐MRI velocity measurements to capture spatially and temporally distributed flow characteristics allows using the Navier‐Stokes equations to estimate the blood pressure gradient.

The available methods of pressure gradient reconstruction from PC‐MRI can be classified in two groups:

- **Direct estimation methods** compute the pressure gradient or difference directly from the fully resolved velocity data. The classical method is to obtain a pressure Poisson equation (PPE) by taking the divergence of the Navier‐Stokes equations and inserting the velocity measurements in the right‐hand‐side.^9^ More recently, several additional methods have been introduced, see a comprehensive review in Bertoglio et al.^10^ In particular, the STE method,^11^ using a Stokes equation by including an auxiliary, nonphysical velocity field, and the WERP method,^12^ based on an integral energy balance of the Navier‐Stokes equation, was presented. These methods are computationally less expensive than solving the Navier‐Stokes equation but require full 3D measurements, the acquisition of which is prohibitive in the clinical practice due to large scan times and the rare availability of 3D PC‐MRI sequences. It is also important to remark that the performance of such *data‐driven* methods is strongly dependent on the image resolution and is susceptible to noise and image artifacts.^10^
- **PDE‐constrained optimization methods** require additionally to 2D or 3D velocity data the anatomy of the vessel. An inverse problem is then formulated where unknown model parameters of the Navier‐Stokes equations, typically as boundary conditions, are computed by minimizing the discrepancy between the numerical solution and the velocity measurements, cf, for instance, previous studies.^13^, ^14^, ^15^, ^16^, ^17^, ^18^ This methodology can handle partial 2D PC‐MRI measurements, which are routinely available in clinical practice. The cost is a higher computational complexity of the inverse problem with respect to the direct estimation methods from 3D data, since several solutions of the Navier‐Stokes equations are required. It furthermore offers a high robustness to measurement noise and resolution, but the quality of the results depends largely on the fidelity of the flow model.

**Figure 1:**
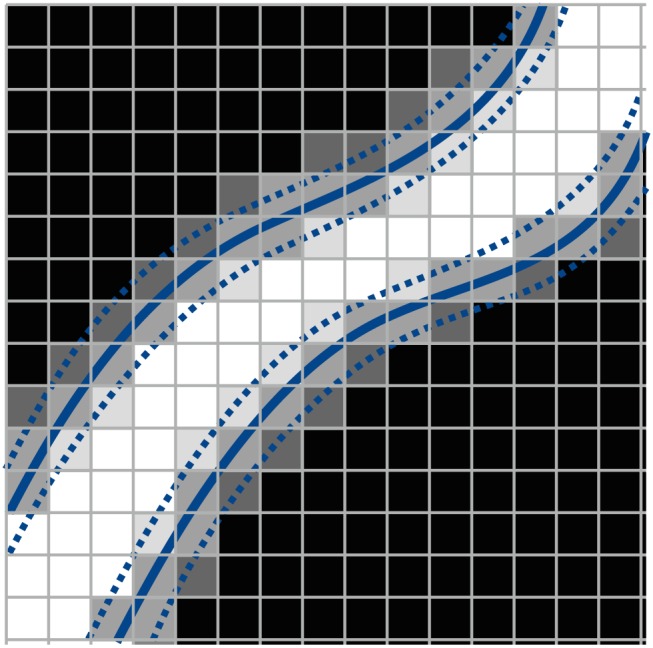
Illustration of potential segmentation errors in a medical image

In this work, we study the performance of the PDE‐constrained optimization approach from 2D PC‐MRI in numerical test cases when geometric errors in the reconstructed 3D domain are present. In cardiovascular modeling, geometry errors arise unavoidably from the segmentation of anatomical medical images (ie, CT or MRI), which are of limited resolution, contain measurement noise, include partial volume effects.^19^, ^20^ Figure 1 illustrates this issue with white pixels denoting interior and black pixels exterior regions of a blood vessel. The separation between both is blurred due to the aforementioned imaging limitations (gray pixels). The blue lines mark possible segmentations of the vessel wall.

The problem of geometric errors in blood flow computations has been recognized and studied previously by Moore et al.^19^, ^20^ Uncertainty quantification studies of geometric uncertainty in CT‐based hemodynamics simulations were subsequently presented in a series of papers.^21^, ^22^, ^23^, ^24^ Recently, theoretical error bounds were derived for finite element discretizations of PDEs under geometric uncertainties.^25^ To the authors' best knowledge, other than improvements to the image segmentation process, no methods have been reported to cope for these inaccuracies.

In this work, we introduce a flow reconstruction methodology which considers alternative slip/transpiration boundary conditions estimated from velocity data, which are able compensate the geometric errors. The methodology is detailed in Section 2. In Section 3, the method is tested in numerical experiments. The results are discussed in Section 4, followed by conclusions in Section 5.

## 2 METHODOLOGY

### 2.1 Fluid flow model

#### 2.1.1 Geometry definitions

**Figure 2:**
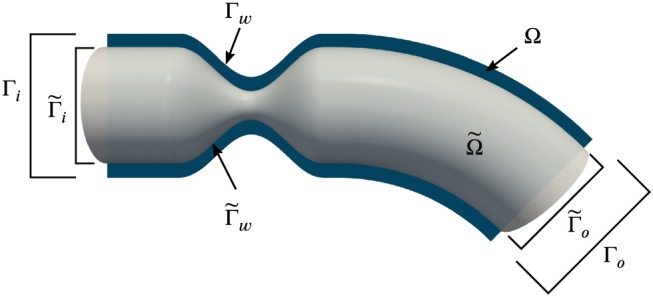
“Approximate” segmented domain $\tilde{\Omega }$ (gray) and cut plane of true domain Ω (blue). $\Gamma_{i},{\tilde{\Gamma }}_{i}$ are proximal to the heart, $\Gamma_{o},{\tilde{\Gamma }}_{o}$ distal. $\Gamma_{w},{\tilde{\Gamma }}_{w}$ denote the vessel wall

Assume that an approximation of the geometry of a blood vessel is obtained by segmenting medical images. We consider both the true geometry and the segmented, approximate version. The true domain of the vessel is denoted by Ω, such that ∂Ω  =  Γ_w_∪Γ_i_∪Γ_o_, with Γ_w_ representing the true vessel wall. The segmented domain is denoted by $\tilde{\Omega }$ and bounded by $\partial \tilde{\Omega }={\tilde{\Gamma }}_{w}\cup {\tilde{\Gamma }}_{i}\cup {\tilde{\Gamma }}_{o}$. Both the true and the segmented domains of a sample vessel are illustrated in Figure 2.

#### 2.1.2 The incompressible Navier‐Stokes equations

Restricting the analysis to large vessels and neglecting elastic effects of the vessel walls, the unsteady Navier‐Stokes equations of an incompressible, Newtonian fluid^26^ are a suitable model to compute the blood flow inside the vessel Ω (and therefore also valid in $\tilde{\Omega }$),

(1a) $$\rho \frac{\partial u}{\partial t}+\rho (u\cdot \Delta )u+\Delta p-\mu \Delta u=0\;\text{in}\;\;\Omega$$

(1b) Δ·u=0inΩ

(1c) $$u(0)=u_{0}\;\;\;\text{in}\;\;\Omega$$

(1d) $$u=g_{d}(x,t)\;\;\;\text{on}\;\;\Gamma_{i}$$

(1e) $$n\cdot \left[\mu \Delta u-1p\right]=g_{n}(x,t)n\;\text{on}\;\;\Gamma_{o}$$

with the velocity vector $u:\Omega \to R^{3}$, the pressure p:Ω→R, the density *ρ* and dynamic viscosity *μ*. Γ_*i*_ denotes inflow boundaries, where the velocity profile **g**_*d*_(**x**,*t*) is specified by means of a Dirichlet boundary condition. Boundary patches denoted by Γ_*o*_ are those where Neumann boundary conditions are given. As boundary conditions for the vessel walls, Γ_*w*_ and ${\tilde{\Gamma }}_{w}$, two models will be used in this work, which are detailed in the following sections.

#### 2.1.3 No‐slip boundary conditions

The most used wall boundary condition is the no‐slip condition, namely,

$$u=0\;\text{on}\;\Gamma_{w}\;\text{or}\;{\tilde{\Gamma }}_{w}.$$

In the remainder of this work, we will assume that this is the correct boundary condition at the true vessel wall Γ_*w*_. We will study the errors which no‐slip boundary conditions on ${\tilde{\Gamma }}_{w}$ induce in the results computed in the approximate geometry $\tilde{\Omega }$.

#### 2.1.4 Slip/transpiration boundary conditions

**Figure 3:**
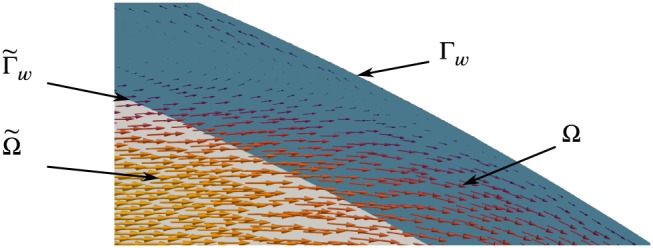
Sketch of slip and transpiration at a virtual boundary ${\tilde{\Gamma }}_{w}$ of the domain $\tilde{\Omega }$, embedded in a “physical” domain Ω with the physical boundary Γ_w_

If the boundaries ${\tilde{\Gamma }}_{w}$ reside inside of the flow region, ie, $\tilde{\Omega }\subset \Omega$, it may be more appropriate to allow for some slip along and transpiration (leakage) across the wall. This situation is illustrated in Figure 3, where a virtual boundary, ${\tilde{\Gamma }}_{w}$, is immersed in the fluid region Ω.

Robin‐type boundary conditions on such artificial domain boundaries allow for flow in wall‐normal and tangential directions, controlled by coefficients, which in the general case may vary in space and time. The coefficients can be defined in such a way that the solution is equal to the corresponding portion of the solution computed on the complete domain with no‐slip conditions on the “true” wall. These boundary conditions, which we refer to as slip/transpiration conditions, can be written in the following form, separating the contributions in the normal and in the tangential directions:

(2a) $$\sum_{k=1}^{d-1}n\cdot \left[\mu \Delta u-1p\right]\cdot t_{k}+\gamma u\cdot t_{k}=0\;\text{on}\;{\tilde{\Gamma }}_{w}$$

(2b) $$\;n\cdot \left[\mu \Delta u-1p\right]\cdot n+\beta u\cdot n=0\;\text{on}\;{\tilde{\Gamma }}_{w}.$$

Here, **n** denotes the outward unit normal vector and **t**_*k*_,*k*  =  1,…,*d* − 1 are orthogonal unit tangent vectors. The number *d*  ∈  {2;3} denotes the geometric dimension of the problem.

Equation 2a is a *slip‐friction* (also called Navier‐slip) boundary condition, see, eg, John and Liakos.^27^ The coefficient *γ* controls the ratio the of tangential stress to the tangential velocity. For *γ*  =  0, this boundary condition is equal to a free slip condition. In the limit *γ*→*∞*, the no‐slip boundary condition (for the tangential velocity component) $\sum_{k=1}^{d}u\cdot t_{k}=0$ is recovered. The transpiration boundary condition, Equation 2b, allows for flow perpendicular to the wall. The amount of transpiration through the wall is controlled by the parameter *β*. The limit *β*→*∞* approaches no‐penetration boundary conditions. In the case of *β*  =  0, the fluid is allowed to freely pass through the wall in normal direction. Both conditions can be set independently, for instance, a free‐slip condition in the tangential directions and a no‐penetration condition for the normal velocity component. In particular, *γ*  =  *β*  =  0 characterizes a free outflow condition, whereas *γ*,*β*→*∞* asymptotically recovers no‐slip boundary conditions. Hence, the combined slip/transpiration boundary conditions are able to represent very different types of boundary conditions, depending only on the coefficients *β* and *γ*. A theoretical analysis of slip/transpiration boundary conditions in the context of the finite element method was presented in John.^28^

For cases where an analytical solution to the Navier‐Stokes equations is known, the parameters can be determined exactly. In Appendix A, the slip model is applied to a Poiseuille flow and the slip parameter computed. Note that in the general case, the values of these coefficients are unknown. Estimating *β* and *γ* from velocity measurements is the subject of Section 2.2.

Note that while our physical justification of the slip/transpiration boundary conditions is based on the assumption $\tilde{\Omega }\subset \Omega$, the model can also be applied to cases where the assumption is violated. However, large improvements in accuracy over no‐slip boundary conditions cannot be reasonably expected. Here, we limit our study to cases where the assumption is valid.

#### 2.1.5 Fractional step scheme

For the sake of computational efficiency, in particular, since solving the inverse problem requires flow computations for several parameter combinations, we employ a fractional step scheme, splitting the original coupled system (1a) to (2b) into a sequence of decoupled, easier to solve PDEs. In particular, we use a version of the classical Chorin‐Temam nonincremental pressure correction scheme.^29^

The method is given in linearized, time semi‐discretized form in Algorithm 1, for the case where slip/transpiration boundary conditions are applied on the boundary patch ${\tilde{\Gamma }}_{w}$.

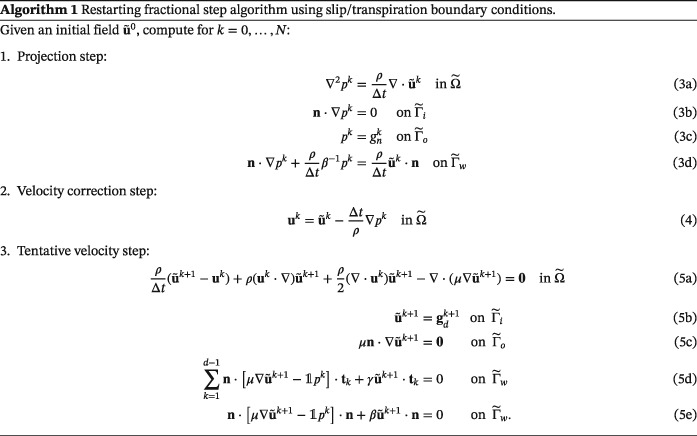

Note that the algorithm is stated for the segmented domain, $\tilde{\Omega }$ with $\partial \tilde{\Omega }={\tilde{\Gamma }}_{w}\cup {\tilde{\Gamma }}_{i}\cup {\tilde{\Gamma }}_{o}$. For the reference domain, simply replace $\tilde{\Omega }$ by Ω and the boundaries ${\tilde{\Gamma }}_{*}$ by Γ_∗_. No‐slip boundary conditions can be defined on ${\tilde{\Gamma }}_{w}$ (or Γ_*w*_) by replacing Equations 5d to 5e by the condition ${\tilde{u}}^{k+1}=0$ on ${\tilde{\Gamma }}_{w}$ (or Γ_*w*_).

Note further that the algorithm starts with the projection and velocity correction steps instead of the tentative velocity step due to the fact that the pressure is required by the slip/transpiration conditions in the tentative velocity step. The given formulation is also convenient with regard to the optimization problem introduced in the subsequent section, since an iteration of the algorithm depends only on the previously computed tentative velocity, representing the *state variable* of the system. Optionally, steps 1 and 2 (computationally inexpensive compared with step 3) of the algorithm can be repeated at the end of each iteration to obtain *p* ^*k* + 1^ and **u** ^*k* + 1^ for postprocessing purposes.

The slip/transpiration boundary conditions appear in both the tentative velocity step, Equations 5d and 5e, and in the pressure projection step, Equation 3d. In the tentative velocity step, the slip/transpiration conditions are treated semi‐implicitly with implicit velocity and explicit pressure from the previous time step. In the pressure projection step, while the slip part does not contribute, the transpiration boundary condition can be expressed via a Robin condition for the pressure with implicit treatment of the velocity and the pressure. This Robin condition, Equation 3d, is derived by considering the normal projection of the velocity correction Equation 4 and rearranging,

(6) $$n\cdot \Delta p^{k}=\frac{\rho }{\Delta t}({\tilde{u}}^{k}-u^{k})\cdot n\;\text{on}\;{\tilde{\Gamma }}_{w}.$$

Assuming that the corrected velocity **u** ^*k*^ and the unknown pressure *p* ^*k*^ satisfy the transpiration boundary condition,

(7) $$n\cdot \left[\mu \Delta u^{k}-1p^{k}\right]\cdot n+\beta u^{k}\cdot n=0\;\text{on}\;{\tilde{\Gamma }}_{w},$$

we can replace **u** ^*k*^·**n** in (6) by (7), assuming *β*  >  0 and obtain

$$n\cdot \Delta p^{k}=\frac{\rho }{\Delta t}\left({\tilde{u}}^{k}\cdot n-\beta^{-1}\left(p^{k}-n\cdot \mu \Delta u^{k}\cdot n\right)\right)\;\text{on}\;{\tilde{\Gamma }}_{w}.$$

As is usual in fractional step methods applied to blood flows,^30^, ^31^ we neglect the viscous term. This results in the final form in Equation 3d. A similar discretization scheme was presented in Caiazzo et al^32^ in the context of immersed porous interfaces.

Note that the implicit treatment of the velocity in the slip/transpiration condition in the tentative velocity step avoids the need of a (in practice very restrictive) stability criterion on the time step. This is particularly reasonable in the context of the Chorin‐Temam method, where additionally very small time steps can cause spurious pressure oscillations if equal order.

### 2.2 The parameter estimation problem

#### 2.2.1 Formulation and solution method

Let us introduce the following short‐hand notation for the discretized numerical model,

$$X_{k}=A_{k}(X_{k-1},\theta ),$$

where $A_{k}$ is the model operator. In the case of the fractional step Algorithm 1 given in Section 2.1.5, the state corresponds to the discrete tentative velocity, $X_{k}:={\tilde{u}}_{h}^{k}\in R^{n}$ and $A_{k}:R^{n}\times R^{p}\mapsto R^{n}$ represents one time iteration of the discrete fractional step scheme. The physical parameters related to the boundary conditions are summarized in $\theta \in R^{p}$, *p*  ≥  1 denoting the number of parameters.

The aim of this work is to estimate *θ* from a sequence of *N* partial velocity measurements $Z_{k}\in R^{m}$, *k*  =  1,…,*N* by means of PDE‐constrained inverse problem. Here, we assume that the measurements are related to the (true) state variable $X_{k}^{t}\in R^{n}$ of the fluid model by means of a measurement operator $H:R^{n}\mapsto R^{m}$, such that

$$Z_{k}=HX_{k}^{t}+\zeta ,$$

where $\zeta \in R^{m}$ represents uncertainty due to measurement errors. The superscript *t* in $X_{k}^{t}$ indicates the ground truth, whereas *X*_*k*_ refers to the state computed by the numerical model.

For the inverse strategy, we adopt a Bayesian estimation approach, where the a priori probability distribution of the parameters is corrected by using the measurements and the model. Assuming that both the probability distribution of the noise and the a priori parameters are Gaussian, the solution of the inverse problem reads: find

(8) $$\begin{array}{ccc} \hat{\theta } & =\underset{\theta }{\text{arg min}}J(\theta ), & \\ J(\theta ) & =\frac{1}{2}{\left\|\theta -\theta_{0}\right\|}_{P_{0}^{-1}}^{2}+\sum_{k=1}^{N}\frac{1}{2}{\left\|Z_{k}-HX_{k}(\theta )\right\|}_{W^{-1}}^{2}. \end{array}$$

*θ*_0_ is an initial guess for the parameters and *P*_0_ the associated covariance matrix. *W* is the covariance matrix associated to the measurement noise.

In this work, we solve problem (8) approximately with the Reduced‐order Unscented Kalman Filter (ROUKF), described in Moireau and Chapelle^33^ and Bertoglio.^34^ It has the advantage of being derivative‐free hence well adapted to complex solvers, including multiphysical problems. It allows also to be flexibly adapted to different discretization strategies. Moreover, as an inherent property of the Kalman filter approach, the parameters are estimated recursively over time and therefore there is no need to store the full dynamic solution as in adjoint‐based methods. The number of forward solutions grows linearly with the number of parameters to be estimated, but the forward solves can be parallelized since they are independent of each other. The ROUKF has become very popular in cardiovascular modeling in general and in particular in computational hemodynamics, see, eg, other works^15^, ^35^, ^36^, ^37^, ^38^

The estimation procedure consists of the following steps: given a sequence of measurements and an approximation of the vessel geometry,

1. estimate the boundary coefficients with the ROUKF,
2. solve the forward problem with the optimized parameters,
3. postprocess the optimized velocity and pressure solution of the forward problem.

#### 2.2.2 Parameters

The inlet velocity (to be set via a Dirichlet boundary condition) is a priori unknown. In this work, we assume a pulsating plug flow,

$$g_{d}(x,t)=-Ūnf(t),$$

where Ū is the velocity amplitude and **n** is the outward normal vector at the boundary. *f*(*t*) is the waveform of the temporal oscillation, for instance,

$$f(t)=\sum_{k=1}^{M}a_{k}\sin(\omega_{k}t),\;a_{1}=1.$$

The amplitude Ū is an unknown constant and needs to be recovered by the parameter estimation procedure. The waveform can easily be estimated prior to solving the inverse problem by postprocessing the measurements. Different parameterizations than the one given are possible. It is assumed here that *f*(*t*) is known beforehand. In practice, a simple approach to obtain the waveform is computing the spatial mean of the velocity data given at the inlet boundary (assuming there are measurements at the inlet) for every measurement time and fitting the time profile. Otherwise, for some chosen small value of *M*, *a*_*k*_ and *ω*_*k*_ can be included in the parameter estimation.

If the slip/transpiration wall‐model is used, the corresponding coefficients *β* and *γ* need to be estimated and are included in the parameter vector.

Summarizing, the parameter vector *θ* consists of the following boundary parameters:

- inflow condition, plug flow parameter Ū,
- slip parameter *γ* (if slip/transpiration BC, per boundary patch),
- transpiration parameter *β* (if slip/transpiration BC, per boundary patch).

## 3 SETUP OF THE NUMERICAL EXPERIMENTS

Numerical experiments are conducted with the goal of comparing the slip/transpiration approach with standard no‐slip boundary conditions in cases where geometric errors are present in the vessel wall. Three realistic synthetic test cases are analyzed, representing arteries with different degrees of stenoses. The setup of the test cases and the numeric solvers used for the forward and the inverse problems are explained in this section.

### 3.1 Geometries

Three geometries with different obstruction ratios of the stenosis of 40%, 50%, and 60% are considered. The latter case is illustrated in Figure 2. The study is conducted under the assumption $\tilde{\Omega }\subset \Omega$, where the slip/transpiration boundary conditions have a sound physical justification. For each stenosis, three computational domains are generated: a reference domain with radius *R*  =  10 mm in the unconstricted parts, which is considered the true domain, and two domains with the outer vessel walls shifted inward by Δ  =  1 mm and Δ  =  2 mm. These offsets are considered segmentation errors with respect to the reference, due to uncertainty—eg, limited resolution (of the order of Δ) and noise—in the medical images. In addition to the reference domain, Figure 2 shows the *approximate* domain for Δ  =  2 mm. In this case, the difference in the radius is 20% in the unconstricted sections, whereas in the throat of the stenosis with 60% obstruction ratio, the radius is halved due to the errors in the geometry.

The true domain, Ω, is used to compute a reference solution for comparison with the estimation framework and to generate synthetic measurements. We pretend that for the pressure drop estimation, this true domain is unknown, but that one of the *approximate* domains is available ( $\tilde{\Omega }$).

### 3.2 Reference solution

#### 3.2.1 Configuration

The reference solution is obtained by solving the fractional step system in the true domain Ω, with no‐slip boundary conditions imposed on the lateral walls Γ_*w*_. At the distal boundary, Γ_*o*_, intersecting the flow, a homogeneous Neumann boundary condition is used, ie, *g*_*n*_  =  0 in Equations 3c and 5c. On the proximal boundary, Γ_*i*_, a pulsating plug flow profile is set via a Dirichlet boundary condition,

$$g_{d}(x,t)=-Ūn\sin(\omega t).$$

Note that **u**  =  **0** on Γ_*i*_∩Γ_*w*_ due to the no‐slip boundary conditions. As above, **n** denotes the outward normal vector on the boundary. To mimic physiologically relevant conditions, we set *ω*  =  2.5*π*s^−1^ and consider the time interval *t*  ∈  [0s,0.4s], approximating the first half of a cardiac cycle, with the peak systole at *t*  =  0.2 s. The viscosity of blood (treated as a Newtonian fluid) is *μ*  =  0.035 g/(cm/s) and the density *ρ*  =  1g/cm^3^. The amplitude of the pulsating inflow velocity is set to Ū=43.75 cm/s, resulting in a peak Reynolds number based on the inlet of $Re=\frac{\rho 2ŪR}{\mu }=2500$. The Reynolds numbers based on the throat of the stenoses, *Re*_*s*_, at the time of peak systole is (obtained from the solution presented below) are listed in Table 1.

**Table 1:** Reynolds numbers at peak systole based on the maximum velocity

Obstruction Ratio	40%	50%	60%
*Re*_*s*_	4063	4863	6055

#### 3.2.2 Discretization and numerical solution

The partial differential equations that constitute the fractional step scheme (3a) to (5e) are discretized in space with the finite element method, using $P_{1}/P_{1}$ basis functions for the velocity and the pressure on an unstructured tetrahedral mesh. Furthermore, streamline‐diffusion stabilization is used with the formula for the stabilization parameter given in Bazilevs et al.^39^ Since backflow is likely to occur at the outflow boundary, velocity‐penalizing backflow stabilization^40^ is added on Γ_*o*_. Note that also transpiration boundary conditions allow for inflow to occur, and therefore, instabilities could potentially arise. In our numerical examples, however, we did not observe such problems, probably since the values of the transpiration parameter are high enough to control that advective energy. However, in case they appear, additional backflow stabilization terms could be added.^40^ In particular, the tangential regularization method presented in Bertoglio and Caiazzo^41^ would be the most suitable since it has shown to be the least intrusive for the pressure field.^40^

The meshes use a reference cell size of *h*  =  0.25*mm* and consist of 3 086 306 to 3 606 417 tetrahedrons and 561 761 to 655 858 vertices, depending on the geometry. The constant time step size is Δ*t*  =  1*ms*.

The solver is implemented using the finite elements library FEniCS.^42^ Preconditioned Krylov methods are used to solve the linear systems, provided by the PETSc package.^43^ We make use of the fact that in the case of no‐slip boundary conditions, the velocity components are completely decoupled in the discretized versions of Equations 4 and 5a and solve three smaller problems for each component separately with the same system matrix, instead of one large system for the complete velocity vector. For solving the tentative velocity equation, we use BICGSTAB preconditioned with diagonal scaling. The pressure Poisson equation is solved with the CG method in the no‐slip case and GMRES if slip/transpiration boundary conditions are used, in both cases with an algebraic multigrid preconditioner. The velocity correction system is solved using CG with a diagonal scaling preconditioner (cf Saad^44^).

### 3.3 Inverse solutions

#### 3.3.1 Measurements

Synthetic partial measurements are generated from the reference solutions in such a way that the measurements are representative for typical 2D PC‐MRI images. This means that 2D planes, intersecting with the 3D domain, are chosen on which the velocity is measured in one specified direction **d**. That is, the measurement is a scalar projection

c=u·d,|d|=1.

**Figure 4:**
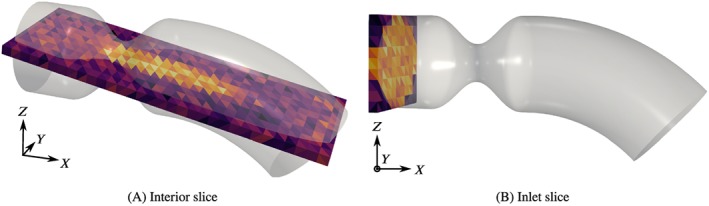
Measurement slices with reference geometry (60%) at the peak time t  =  0.2s, with resolution H  =  2m m

Since the inflow velocity is unknown and needs to be estimated, one plane will be placed at the inlet (Figure 4A). We consider here the *x* velocity component, orthogonal to the plane. A second plane intersects the domain lengthwise with an inclination of ≈10*ř* with respect to the *xz*‐plane. It connects points at the inlet, in the throat of the stenosis and at the outlet, as shown in Figure 4B. The velocity component is chosen tangential to the plane in the streamwise direction (ie, parallel to the longer edge).

These slices have a finite thickness and consist in one layer of 3D voxels. The measurement data are represented on a mesh of uniform, equally sized tetrahedra. The thickness of the slices equals the element edge length on the plane, *H*. The element length is chosen to match typical voxel sizes for PC‐MRI, namely, *H*= 1 and 2 mm. We limit this study to the cases where the geometry error Δ is equal to the voxel size of the measurements, supposing that the same hypothetical image resolution was used to obtain the 3D vessel geometry and the PC‐MRI velocity images. We refer to the case Δ  =  *H*  =  1*mm* as “Δ_1_” and Δ  =  *H*  =  2*mm* as “Δ_2_.”

The measurements are obtained by interpolating the selected component of the reference velocity to the barycenters of the tetrahedra of the slice meshes. The measurement data are considered constant within each tetrahedron, as can be seen in Figure 4 for noisy example data. The temporal sampling of the measurements is Δ*T*  =  20*ms*, representing a typical value for 2D‐PCMRI.

The noise intensity in the velocity data in PC‐MRI is proportional to the *VENC* parameter of the scan, which encodes the intensity of the velocity encoding magnetic gradients.^45^, ^46^ Therefore, in practice, the *VENC* is chosen as small as possible to reduce the noise in the velocity image. However, this parameter has to be set for each measurement sequence to a value higher than the expected maximum velocity in order to avoid velocity aliasing.^45^, ^46^ Since the *VENC* is fixed for the entire duration of a MRI scan, the noise level in all voxels is proportional to the global maximum velocity in space and time in the measurement region, regardless of the measured instantaneous local velocities. It is therefore realistic to assume that in practice, in order to improve the velocity‐to‐noise ratio, different values of the *VENC* parameter would be used for the different slices, according to the anticipated flow conditions. In the clinical practice, it can be expected that high‐quality acquisitions contain a velocity noise of 10% of the peak velocity.^8^

Therefore, in the numerical experiments presented here, Gaussian white noise is added to each of the slices independently with a standard deviation of 15% of the maximum velocity of the reference solution in the measurement region. Table 2 lists the values of the maximum velocities of the reference configurations (the complete results are presented in Section 4.1) and the corresponding measurement noise intensities in terms of the standard deviation for the inlet slice and the interior slice with different coarctation ratios of the stenosis.

**Table 2:** Maximum velocities and standard deviation of Gaussian noise at the inlet and in the interior image slices, for different obstruction ratios of the stenosis

	Inlet Slice		Interior Slice	
	All Stenosis	40% Stenosis	50% Stenosis	60% Stenosis
maxU	43.75 cm/s	140 cm/s	200 cm/s	320 cm/s
*σ*_noise_	6.56 cm/s	21 cm/s	30 cm/s	48 cm/s

#### 3.3.2 Forward solution

The optimization procedure requires evaluations of the forward model, ie, the fractional step algorithm. The configuration of the forward model and solvers is identical to the reference simulations, with the following exceptions:

- the “approximate” computational domains $\tilde{\Omega }$ with geometric errors are used,
- no‐slip or slip/transpiration boundary conditions on ${\tilde{\Gamma }}_{w}$,
- boundary parameters are unknown and estimated (see the next paragraph).

Note that using slip/transpiration boundary conditions in implicit form, the velocity components in the momentum Equation 5a are coupled and cannot be solved for separately. This results in an increase in CPU time compared with the no‐slip case. In the case of slip/transpiration boundary conditions, the momentum equation is solved with GMRES, preconditioned with algebraic multigrid. With no‐slip boundary conditions the same solvers are used as for the reference solution, see Section 3.2.2.

#### 3.3.3 Physical model parameters

We compare two wall models:

1. standard no‐slip boundary conditions and
2. slip/transpiration boundary conditions.

The only parameter of the no‐slip model is the plug flow parameter at the inlet. It seems therefore reasonable to estimate the plug flow parameter only from measurements given at the inlet. Regarding the geometric errors, it will be examined if the results can be improved by providing additional measurements in the interior of the domain, ie, by using both measurement slices discussed above. In the case of slip/transpiration boundary conditions, measurements at the inlet and in the interior will be used in order to estimate the plug flow parameter and the boundary coefficients *β* and *γ*.

Summarizing, the parameters to be estimated are the following:

- **no‐slip**
  θ=Ū,
- **slip/transpiration**
  $$\theta =\left(Ū,\;\beta ,\;\gamma \right).$$

#### 3.3.4 Kalman filter parameters

The physical parameters to be estimated (see paragraph above) are reparameterized as $\theta^{\prime }=\log_{2}(\theta )$. By optimizing *θ* ^*′*^, it is ensured that the physical parameters *θ*, which enter the fluid model, stay positive. This is required to guarantee the positivity of the variational formulation of the forward problem and in agreement with basic physical intuition, since, for instance, with a negative slip parameter the wall‐tangential flow would be accelerated by the traction, instead of slowed.

Initial guesses for the parameters and the associated uncertainties have to be provided for the ROUKF algorithm. We choose

$$\theta_{0}=\left\{\begin{array}{c} 40\\ 0.001\\ 5000 \end{array}\right\}\;\begin{array}{ccc} & \text{plug flow,} & \\ & \text{slip parameter,}\\ & \text{transpiration parameter.} \end{array}$$

The initial variances of the reparameterized parameters *θ* *′* are set to $\sigma_{0}^{2}=1$. The weights *W* in (8), representing the uncertainty in the measurements, is set to the known noise intensity in each of the slices, ie, *W*  =  diag(*σ*), with $\sigma \in R^{m}$ the vector of the noise standard deviations in all *m* measurement data points. In practice, *σ* is the estimated noise level proportional to the VENC value used for each measurement.

### 3.4 Summary

The cases included in this study are summarized in Table 3. In total, 540 optimization problems with subsequent forward simulations with each optimized set of parameters are solved. Each simulation is computed on 16 Intel Xeon 2.5 GHz cores on the Peregrine HPC cluster of the University of Groningen.

**Table 3:** Summary of numerical experiments

Model	Obstruction Ratio	Measurement Slices	Δ, *H*	Parameters	Random Samples
No‐slip	{40%, 50%, 60%}	inlet only	{Δ_1_,Δ_2_}	Ū	30
No‐slip	{40%, 50%, 60%}	inlet + interior	{Δ_1_,Δ_2_}	Ū	30
Slip/transpiration	{40%, 50%, 60%}	inlet + interior	{Δ_1_,Δ_2_}	Ū, *β*, *γ*	30

## 4 NUMERICAL RESULTS

The results obtained with no‐slip and with slip/transpiration boundary conditions are mainly analyzed in terms of the pressure drop and the velocity error. The pressure drop is defined as the difference in the pressure averages at two cross‐sections, upstream and downstream of the stenosis,

(9) $$\delta p^{k}=\frac{1}{\left|\Gamma_{i}\right|}\int_{\Gamma_{i}}p^{k}-\frac{1}{\left|\Gamma_{o}\right|}\int_{\Gamma_{o}}p^{k},$$

with $\left|\Gamma_{⋄}\right|$ denoting the area of a boundary patch and the superscript *k* the *k*th time step. Note that the pressure drop is determined by the pressure gradient alone and does not depend on fixing the pressure constant. The velocity error is considered in the *L* ^2^‐norm over the whole approximate domain, scaled by the global maximum velocity, and defined as

(10) $$E^{k}:=\frac{\|{û}^{k}-Iu^{k}{\|}_{L^{2}(\tilde{\Omega })}}{\max_{k}\|Iu^{k}{\|}_{L^{2}(\tilde{\Omega })}}.$$

Here, I is the operator which interpolates the reference velocity **u** ^*k*^ to the space of the optimized velocity ${\hat{u}}^{k}$, ie, from the reference geometry Ω to the approximate geometry, $\tilde{\Omega }$.

We proceed by first presenting the numerical solutions of the reference setups, followed by a discussion of the results of the inverse problems using no‐slip boundary conditions on the walls. Lastly, we present the results of the slip/transpiration model and compare them with the no‐slip results.

### 4.1 Reference solution and measurements

We briefly discuss the numerical solutions of the reference cases. These form the basis of the subsequent analysis of the results of the optimization problems, because they serve as the ground truth which the solutions of the inverse problems are compared to. In addition, the measurements are generated from the velocity solution of the reference, as was explained above.

**Figure 5:**
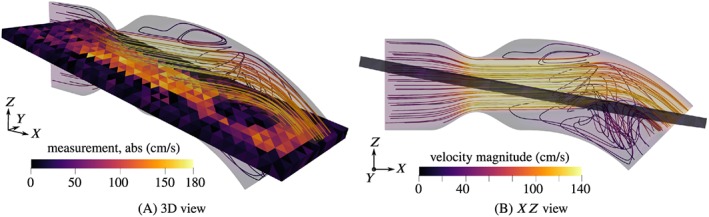
Streamlines of the reference flow and sample noisy velocity measurement (in‐plane component, Δ_2_) at peak systole for 40% obstruction ratio

**Figure 6:**
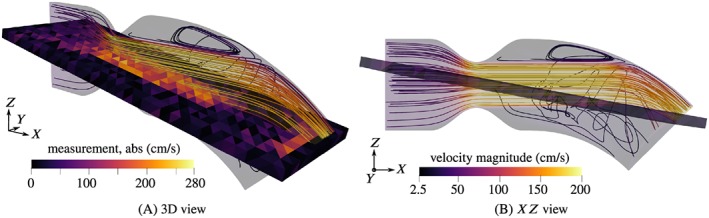
Streamlines of the reference flow and sample noisy velocity measurement (in‐plane component, Δ_2_) at peak systole for 50% obstruction ratio

**Figure 7:**
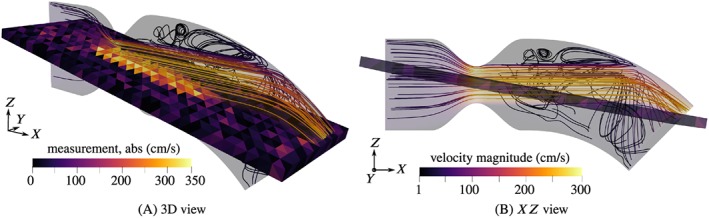
Streamlines of the reference flow and sample noisy velocity measurement (in‐plane component, Δ_2_) at peak systole for 60% obstruction ratio

Streamlines of the velocity field are shown in Figures 5, 6, 7, for peak systole, *t*  =  0.2*s*. The domain is cut along the *XZ* plane and only one half is shown, since the flow is approximately symmetrical with respect to that plane. The figures furthermore include the interior measurement plane with a resolution of *H*  =  2*mm*.

Since the flow is of pulsating character, dynamic effects are very pronounced. We restrict the discussion here to the flow situation at peak systole, *t*  =  0.2*s*, where the maximum velocities and pressure drops can be expected. Round jets are formed due to the constrictions, surrounded by annular recirculation zones. The jets impinge on the curved wall and are mainly deflected towards the outlet. Secondary circulations form in particular below the jets and are fed by azimuthal wall‐bound flow produced by the impingement. In the example with 40% obstruction ratio, this effect is most pronounced. The recirculation velocities are considerable compared with the velocities of the jet, and the strong recirculation bubble acts back on the jet flow by pushing it upward. Such an interaction between recirculation zones and jets does not appear in the cases of more severe stenosis, 50% and 60%, where the jets remain unperturbed. The magnitude of the secondary flow patterns seems negligible in comparison to the very high jet velocities. The snapshot of the measurement of the 40% case, Figure 5A, shows that the recirculation is captured to some degree in the measurements. There is a “dead region” of low in‐plane velocities in the center, near the outlet, surrounded by higher magnitude wall‐bound flow. Such features are not recognizable in the 50% and 60% cases due to the high noise intensity. Weak backflow is present at the outflow boundary in all examples, confirming the need for backflow stabilization.

**Figure 8:**
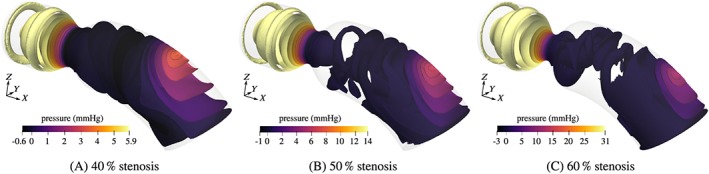
Pressure isosurfaces of reference problems with different coarctation ratios at the peak time t  =  0.2s

Isosurfaces of the corresponding pressure fields are shown in Figure 8. The pressure is close to zero along the outflow boundaries, due to the homogeneous Neumann boundary condition. As the flow accelerates in the stenosis, strong very localized pressure minima appear at the wall in the narrowest section and propagated downstream. The jet impingement creates a region of relatively high pressure in the region of the impact. The maximum pressure is naturally located upstream of the stenosis and distributed rather uniformly. The maximum pressure is highest for the stenosis with 60% obstruction ratio.

### 4.2 Estimation results for the no‐slip model

Consider first the scenario where measurements are given only at the inlet. The PDE‐constrained optimization problem is solved with no‐slip boundary conditions, estimating the plug flow parameter.

Statistics of the plug flow parameters estimated from measurements at the inlet with different resolutions and geometry errors in the computational domain Δ  =  *H*  =  1 and 2 mm are listed in Table 4. Since the ROUKF algorithm optimizes the $\log_{2}$‐reparameterized parameter and assumes *θ* to be normally distributed, a lognormal distribution can be considered for the physical parameters, 2^*θ*^. The table shows the mean and the square root of the variance of the physical nonlogarithmized parameter assuming a lognormal distribution over 30 identical repetitions of the experiment for independent random realizations of measurement noise. The plug flow parameter is recovered with a very good accuracy, with errors of less than 0.5% compared with the ground truth. The variability of the parameter is generally very small, being largest for Δ  =  2*mm* in all investigated obstruction ratios, possibly due to the lower resolution of the measurements and thus less data being available.

**Table 4:** Mean and square root of the variance of the estimated plug flow parameter, using no‐slip BCs and measurements only at the inlet

Δ, mm	40% Stenosis		50% Stenosis		60% Stenosis	
	Mean	$\sqrt{\mathrm{Var}}$	Mean	$\sqrt{\mathrm{Var}}$	Mean	$\sqrt{\mathrm{Var}}$
1	43.98	0.06	43.98	0.06	43.93	0.05
2	43.67	0.15	43.61	0.20	43.71	0.13

*Note*. Statistics from 30 independent realizations of noisy measurements. Ground truth: 43.75 cm/s.

**Figure 9:**
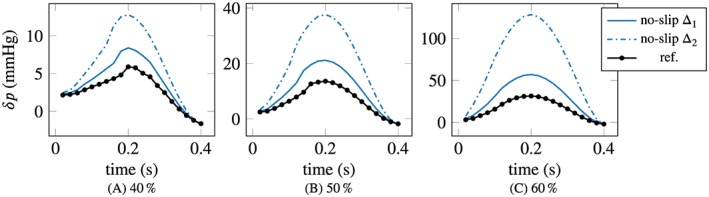
Mean pressure drop with no‐slip BCs for 30 realizations of noise. The peak standard deviation is of the order of 0.1% of the mean. Measurements were given at the inlet with resolution H  =  Δ, Δ denoting the error in the geometry (cf legend); Δ_1_  =  1mm and Δ_2_  =  2mm

The mean pressure drop, obtained by forward‐solving the Navier‐Stokes equations with the optimized parameters, is visualized in Figure 9 over time for the three investigated obstruction ratios and for both geometry errors/measurement resolutions, Δ_1_  =  1mm and Δ_2_  =  2mm. The standard deviation over 30 experiments is below 0.5% of the mean value at peak systole, similarly to the plug flow parameter. This indicates that the procedure is very robust to noise and with respect to small changes in the parameter.

On the other hand, it is immediately evident from the figures that the accuracy of the pressure gradient reconstruction is very poor, especially for large obstruction ratios, when errors in the geometry are present. In the best scenario, the mildest stenosis with 40% obstruction and for Δ_1_ (ie, for the smaller geometry error and measurement resolution Δ  =  *H*  =  1mm), the error in the pressure drop at the peak is about 50%. With Δ_2_ (Δ  =  *H*  =  2*‘*mm), the error exceeds 100%. For the more severe 50% and 60% stenoses, the peak error is of the order of 100% for Δ_1_, and for Δ_2_ rises up to 300% to 400%.

**Figure 10:**
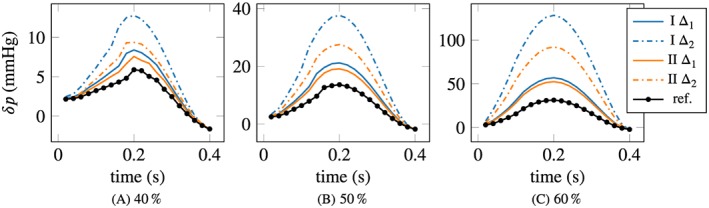
Mean pressure drop with no‐slip BCs for 30 realizations of noise; standard deviation of the order of 0.1% of the mean. Measurements given on two slices (labeled “II”) vs measurements only at the inlet (“I”). Δ_1_  =  1m m and Δ_2_  =  2m m (cf Figure 9)

The pressure drop estimates are improved by taking into account additional measurements in the interior. Figure 10 shows the pressure drops obtained for the case where two measurement slices were used (label “II” in the figure), at the inlet and the lengthwise intersecting slice, in comparison to measurements only at the inlet (label “I,” same curves as in Figure 9). The discrepancy between the model and reference pressure gradient solutions is reduced by a large factor in the case of Δ  =  2mm, and to a lesser degree for Δ  =  1mm. Table 5 compares the corresponding estimated plug flow parameters for the cases with measurements at the inlet (rows labeled “I”) and measurements at the inlet and in the interior slice (“II”). By considering measurements in the interior, the estimated plug flow parameter deviates significantly from the ground truth, compared with inlet‐only measurements, the error is largest for the 60% stenosis with Δ  =  2*mm* with 20% underestimation of the ground truth, compared with 0.1% using only measurements at the inlet. Hence, the improved pressure drop estimation comes at the cost of large errors in the inflow profile.

**Table 5:** Mean and square root of the variance of the estimated plug flow parameter, using no‐slip BCs and measurements only at the inlet

Δ, mm	# Slices	40% Stenosis		50% Stenosis		60% Stenosis	
		Mean	$\sqrt{\mathrm{Var}}$	Mean	$\sqrt{\mathrm{Var}}$ M mean	$\sqrt{\mathrm{Var}}$	
1	I	43.98	0.06	43.98	0.06	43.93	0.05
	II	41.48	0.05	41.58	0.05	41.82	0.06
2	I	43.67	0.15	43.61	0.20	43.71	0.13
	II	37.40	0.11	36.46	0.19	35.06	0.14

*Note*. Statistics from 30 independent realizations of noisy measurements. Ground truth: 43.75 cm/s.

**Figure 11:**
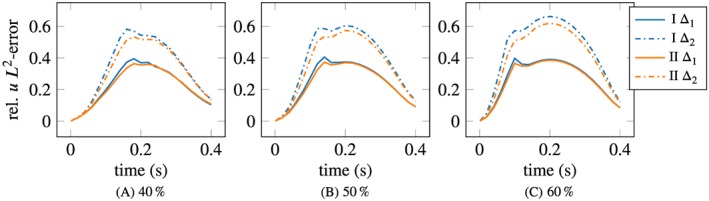
Mean velocity error with no‐slip BCs for 30 realizations of noise; peak systole standard deviation of the order of 0.1% of the mean. Measurements given on two slices (“II”) vs measurements only at the inlet (“I”). Δ_1_  =  1mm and Δ_2_  =  2mm

Figure 11 shows the velocity error, defined by Equation 10, over time. The velocity error globally increases slightly with augmenting obstruction ratios of the stenoses, but to a much lesser degree than the error in the pressure drop. Computations with a bigger geometry error, ie, Δ_2_ instead of Δ_1_, lead to increased errors in the velocity by roughly 50% in all three cases. By taking into account interior measurements (lines labeled “II” in Figure 11), the errors are slightly reduced, especially for Δ_2_.

Again, the results are very robust to noise with relative standard deviations of the velocity error of the order of 0.1% at peak systole.

The observed poor pressure drop estimates and large errors in the inflow velocity render the described procedure using no‐slip boundary conditions inadequate for the application discussed here. The decreased radius in the stenosis gives rise to a much higher pressure drop if the inflow velocity is similar to the reference case. In order to fit interior measurements (for instance the jet velocities), if given, the inflow velocity has to be strongly decreased.

This reasoning motivates investigating slip/transpiration boundary conditions. The results of the numerical experiments using slip/transpiration boundary conditions are presented in the following section.

### 4.3 Estimation results for the slip/transpiration model

**Figure 12:**
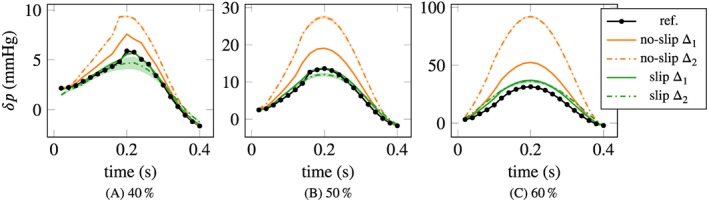
Pressure drop comparison, slip/transpiration (“slip”) vs no‐slip. Mean values with ±2σ bands over 30 samples of measurements, given at the inlet and in the interior plane, with resolution/geometry error Δ_1_  =  1mm and Δ_2_  =  2mm

Consider the case where measurements are given at the inlet and on the interior slice. The pressure drop obtained with the slip/transpiration boundary conditions is displayed in Figure 12 in comparison to the no‐slip results, also considering both measurement slices. The accuracy of the pressure drop estimation is greatly improved in all cases. Especially with Δ  =  1mm, for the 40% and 50% cases, the estimated pressure drop now coincides almost perfectly with the ground truth. In the most severe 60% stenosis, the pressure drop is overestimated by 15% for both Δ_1_ and Δ_2_. Using the Δ_2_ geometry and measurements leads to a slight underestimation of the pressure drop in the 50% example, and to a more pronounced underestimation for the 40% case.

**Figure 13:**
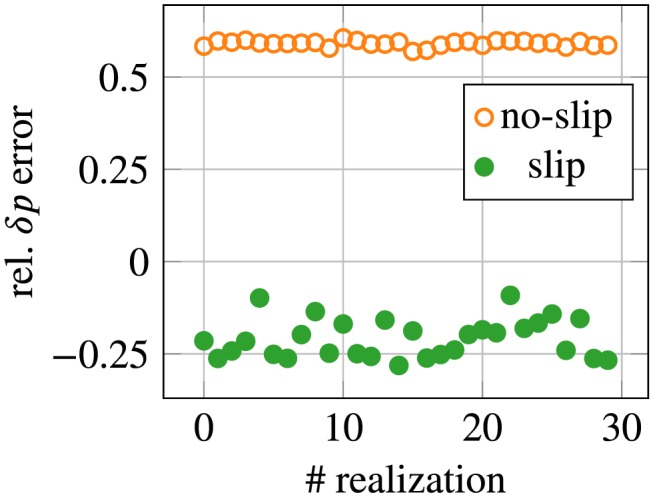
Relative, signed pressure drop error at peak systole compared for no‐slip and slip/transpiration boundary conditions, for 40% obstruction ratio and Δ_2_. Each point corresponds to the result obtained for one realization of noisy measurements

The figure also shows the variability of the pressure drop by means of ±2*σ* bands computed for 30 realizations of noise. The spread seems negligible for all cases except in the setting of the 40% stenosis, using the slip/transpiration model and Δ_2_ measurements, where a larger variability is present in the pressure drop than in the other experiments. Increasing the sample size to 50 for this example did not significantly reduce the variance observed in the pressure drop. Albeit the larger spread with the slip/transpiration model in this particular case, the estimated pressure drop was still observed to be closer to the ground truth in all simulated cases. This is shown in Figure 13, where the error in the pressure drop at peak systole is plotted for the 30 investigated realizations of noisy measurements. The slip/transpiration model underestimates the ground truth by approximately range 10% to 25% whereas the error with the no‐slip model is around 60%.

**Figure 14:**
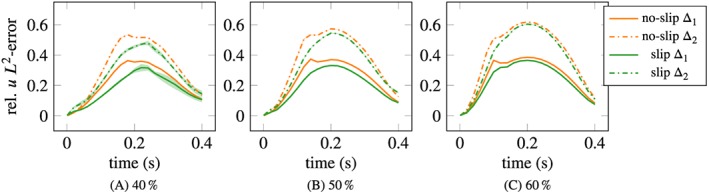
Velocity error comparison, slip/transpiration (labeled “slip”) vs no‐slip. Mean values with ±2σ bands over 30 samples of measurements, given at the inlet and in the interior plane, with resolution/geometry error Δ_1_  =  1mm and Δ_2_  =  2mm

The corresponding relative *L*_2_ velocity errors are shown in Figure 14. In all cases, the error is smaller with the slip/transpiration model, the relative improvement being the most pronounced for 40%. Some variability in the error can be observed after the peak time *t*  =  0.2*s* in the 40% case for both values of Δ, using the slip/transpiration model.

Statistics of the estimated plug flow parameter are compared for both models in Table 6. With slip/transpiration boundary conditions, the ground truth is recovered with very good accuracy for both Δ_1_ and Δ_2_. In all settings, the errors are significantly smaller compared with those obtained with no‐slip boundary conditions. The variability is generally small with the square root of the variance below 1% of the mean. In the case of 40% obstruction ratio with Δ_2_, the square root of the variance is somewhat increased for slip/transpiration conditions, to 2% of the mean. This coincides with the observation of an increased variability in the pressure drop for the 40% case with Δ_2_.

**Table 6:** Mean and square root of variance of the estimated plug flow parameter $\theta_{Ū}$, using slip/transpiration and no‐slip BCs, for 30 independent realizations of noisy measurements at the inlet and in the interior

Δ, mm	Model	40% Stenosis		50% Stenosis		60% Stenosis	
		Mean	$\sqrt{\mathrm{Var}}$	Mean	$\sqrt{\mathrm{Var}}$	Mean	$\sqrt{\mathrm{Var}}$
1	no‐slip	41.48	0.05	41.58	0.05	41.82	0.06
	slip	43.19	0.13	44.46	0.07	44.21	0.08
2	no‐slip	37.40	0.11	36.46	0.19	35.06	0.14
	slip	44.01	1.03	44.80	0.16	45.40	0.13

*Note*. Ground truth: 43.75 cm/s.

For the transpiration and slip parameters no ground truth values are available. The transpiration parameter *β*, summarized by Table 7, increases with the obstruction ratio. The stronger the stenosis and jet, the higher is therefore the resistance to flow across the boundary in the normal direction. The parameter is smaller for Δ_2_ than for Δ_1_, since in the former case the boundaries are located deeper inside the true flow domain and more transpiration has to be permitted. For the 40% case the variance‐to‐mean ratio is larger than for the 50% and 60% geometries.

**Table 7:** Transpiration parameter β. Mean and square root of variance for 30 samples of noisy measurements (inlet & interior slices)

Δ, mm	40% Stenosis		50% Stenosis		60% Stenosis	
	Mean	$\sqrt{\mathrm{Var}}$	Mean	$\sqrt{\mathrm{Var}}$	Mean	$\sqrt{\mathrm{Var}}$
1	6684.48	257.45	8654.17	150.88	20425.33	475.04
2	2075.99	394.95	3573.48	147.31	8413.68	318.07

The slip parameter exhibits a more complex behavior. Its statistics are summarized in Table 8. While under Δ_1_, the mean of the slip parameter is of the same order of magnitude for 40%, 50%, and 60% stenoses, the mean values vary strongly with Δ_2_. Using the 50% and 60% geometries in the Δ_2_ setting the slip parameter is smaller by orders of magnitude than the corresponding values observed with Δ_1_, and tends towards free‐slip conditions. A high variability in the slip parameter is observed for 40%, in accordance with the behavior of the pressure drop and the velocity error. For 50% obstruction ratio, the square root of the variance is much smaller for Δ_1_, only about 10% of the mean value, and high for Δ_2_. In the 60% case, the variance in the parameter is elevated for both Δ_1_ and Δ_2_. In these scenarios, the variability in the pressure drop and the velocity error were seen to be negligible.

**Table 8:** Slip parameter γ. Mean and square root of variance for 30 samples of noisy measurements (inlet & interior slices)

Δ, mm	40% Stenosis		50% Stenosis		60% Stenosis	
	Mean	$\sqrt{\mathrm{Var}}$	Mean	$\sqrt{\mathrm{Var}}$	Mean	$\sqrt{\mathrm{Var}}$
1	0.41	0.36	0.43	0.04	0.24	0.43
2	0.59	0.97	6.23e‐08	4.09e‐07	2.75e‐05	1.08e‐05

The increased variability obtained with the slip/transpiration model in the case of 40% obstruction ratio, compared with the more severe stenoses with 50% and 60%, can most likely be attributed to the more complex recirculating flow patterns in the former case. The wall‐bound, mainly azimuthally circulating flow of the 40% stenosis is very sensitive to the wall parameters. The interior measurement slice, however, contains little information about these flow features, as can be seen in Figure 5A. The optimized slip and transpiration parameters must accommodate principally to the flow in the stenosis, the impingement region of the jet and also the recirculating flow caused by the impingement. In the 50% and 60% cases, the secondary flow patterns seem to be of negligible importance. The wall parameters only have to account mainly for the correct behavior in the stenosis and in the impingement region of the jet.

## 5 CONCLUSIONS

We presented a framework for estimating quantities derived from the hemodynamic pressure and/or velocity, using 2D‐PCMRI velocity measurements, a reconstruction of the blood vessel geometry of interest, and a suitable fluid model. The focus of the analysis was on the effect of errors in the wall position, eg, due to imperfect image segmentation, on the estimated pressure drop in the case of arterial stenosis. Our results are consistent with other studies of the issue of geometric uncertainties. In Moore et al,^19^, ^20^ the importance of geometric errors on MRI‐based hemodynamics was studied for the first time. In previous works,^21^, ^23^, ^24^ uncertainty quantification of CT‐based blood flow simulations showed an important impact of geometric uncertainties on the hemodynamic pressure in stenotic coronary arteries and a strong sensitivity of the wall shear stress with respect to geometry errors in a synthetic carotid artery bifurcation aneurysm.^22^

Also very recently in Minakowski and Richter,^25^ the problem of geometric uncertainty was studied theoretically, providing error bounds and finding a large impact of small boundary variations on the numerical solution. However, to the best of the authors’ knowledge, this is the first time that a methodology for coping for geometric uncertainties is proposed. In order to reduce the errors induced in the pressure drop by using no‐slip boundary conditions on inaccurate vessel walls, we employed slip/transpiration boundary conditions, the coefficients of which were included in the parameter estimation procedure.

Both wall models were compared for synthetic test cases of stenosis with different severities. It was observed that no‐slip conditions imposed on inaccurate walls (ie, shifted with respect to a ground truth) indeed induce huge errors in the estimated pressure drop. Optimized slip/transpiration boundary conditions allowed the temporal evolution of the pressure drop to be estimated with very good precision, and additionally delivered accurate estimates of the inlet velocity. The method proved capable of handling 2D‐PCMRI‐type measurements, ie, a scalar velocity component in a defined direction, on selected pseudo‐2D planes, with realistic, coarse image resolutions and suffering from strong random noise, especially in the regions of low velocities.

In the presented study, the parameters of the slip/transpiration boundary conditions were considered constant over the whole boundary and in time. Allowing for some variation in space and time is likely to further improve the results, especially with regard to more complex realistic geometries and real data.

A limitation of the study was the assumption that the approximate domain was a subset of the true domain. This is the regime where an improved accuracy with slip/transpiration boundary conditions can be reasonably expected. However, the model is also applicable to the case where the assumption does not hold, but a significant reduction of the errors obtained with no‐slip boundary conditions should not be expected. In such a scenario, the slip/transpiration model seems to tend towards no‐penetration/free slip behavior and yields marginally reduced errors in the pressure drop, compared with the no‐slip case.

The methodology is limited to large vessels, where 2D‐PCMRI scans are feasible and the assumption of blood as a Newtonian fluid is reasonable. Elastic deformation of the vessel walls was neglected and combining the slip/transpiration model with fluid‐structure interaction remains a question for future work. Furthermore, the flow conditions are likely to be in the regime of transition to turbulence. It seems worthwhile, especially with regard to real data, exploring the discussed phenomena using turbulence models, ie, large eddy simulation.

## APPENDIX A SLIP BOUNDARY CONDITION FOR THE POISEUILLE FLOW

### A.1

In settings where an analytical solution of the incompressible Navier‐Stokes equations is known, the coefficients of the slip/transpiration boundary conditions can be determined exactly. For instance, consider the simple Poiseuille flow example of steady state flow through a straight tube with constant circular cross‐section. In this situation, the Navier‐Stokes equations simplify to

(A1) $$\frac{1}{r}\left(r\frac{du}{dr}\right)=-\frac{G}{\mu },$$

in cylinder coordinates, where *u* is the axial velocity component. The radial and the angular components are zero. *G* is a constant pressure gradient acting on the fluid in the axial direction. Equation A1 can be solved by assuming a symmetry boundary condition $\frac{du}{dr}=0$ at *r*  =  0, and a no‐slip boundary condition *u*(*r*  =  *R*)  =  0, where *R* is the radius of the tube. The resulting velocity profile along the radial coordinate *r* is given by

(A2) $$u(r)=\frac{G}{4\mu }(R^{2}-r^{2}).$$

Consider now the situation where the (virtual) boundary of the domain is moved away from the wall to *r*  =  *R* *′* in the interior of the tube. Let us pretend that the velocity distribution is unknown, but that we know *R*, *R* *′*, and the fact that *u*(*R*)  =  0. A boundary condition at this virtual boundary *r*  =  *R* *′* can be defined in terms of a Robin condition:

(A3) $$\mu {\left.\frac{du}{dr}\right|}_{r=R^{\prime }}+\gamma u(R^{\prime })=0.$$

The solution of Equation A1 with boundary conditions $\frac{du}{dr}=0$ at *r*  =  0 and the Robin condition (A3) is given by Equation A2 if the proportionality factor *γ* is chosen such that

$$\gamma =2\mu \frac{R^{\prime }}{R^{2}-R^{\prime 2}}.$$

Here, *γ* depends only on the geometry.
